# Exploring the Feasibility of Estimating Intraocular Pressure Using Vibrational Response of the Eye: A Methodological Approach

**DOI:** 10.3390/s24123997

**Published:** 2024-06-20

**Authors:** Seongwook Jeon, Gyungmin Toh, Junhong Park, Won June Lee

**Affiliations:** 1 Department of Mechanical Engineering, Hanyang University, Seoul 04763, Republic of Korea; xingyu0810@hanmail.net (S.J.); avlrudals@gmail.com (G.T.); 2 Department of Ophthalmology, Hanyang University College of Medicine, Seoul 04763, Republic of Korea; 3 Department of Ophthalmology, Hanyang University Seoul Hospital, Seoul 04763, Republic of Korea

**Keywords:** intraocular pressure, vibration, dynamic responses, transfer function analysis, non-invasive measurement

## Abstract

This study addresses the limitations of current tonometry techniques by exploring vibroacoustic properties for estimating intraocular pressure (IOP), a key diagnostic parameter for monitoring glaucoma—a significant risk factor for vision loss. Utilizing vivo porcine eyeballs, we investigated the relationship between IOP and the nonlinear vibration transfer function ratio (NVTFR). Through applying varying vibration levels and analyzing responses with transfer function analysis and univariate regression, we identified a strong negative correlation between NVTFR and IOP, evidenced by a Pearson correlation coefficient of −0.8111 and significant results from generalized linear model (GLM) regression (*p*-value < 0.001). These findings indicate the potential of NVTFR as a vital indicator of IOP changes. Our study highlights the feasibility of using vibroacoustic properties, specifically NVTFR, to measure IOP. While further refinement is necessary for in vivo application, this approach opens new possibilities for non-invasive and patient-friendly IOP monitoring, potentially enhancing ophthalmology diagnostic techniques and providing a foundation for future research and development in this critical area.

## 1. Introduction

In the field of ophthalmology, assessing IOP is a critical routine for eye specialists. Elevated levels of IOP are significantly linked with the onset and advancement of glaucoma [1,2]. Although the risk of glaucoma exists independently of IOP [3], the predominant therapeutic approach focuses on IOP reduction. The relevance of IOP monitoring thus becomes evident in both assessing treatment response and patient follow-up. Two distinct forms of glaucoma, primary open-angle glaucoma and normal-tension glaucoma, are differentiated based on untreated IOP levels: above 21 mmHg for the former and 21 mmHg or below for the latter. IOP monitoring plays a pivotal role in managing glaucoma, particularly in tracking the response to treatments and the progression of the disease.

For IOP measurement, an ideal device is one that provides accurate, consistent, and repeatable results without altering the IOP or causing ocular damage. In the current medical practice, various tonometry methods are in use, including noncontact tonometers, the widely accepted Goldmann applanation tonometer (GAT), rebound tonometers, and the Tono-Pen [4,5,6,7,8,9,10]. The GAT, in particular, is considered the gold standard among tonometers. However, its application can be cumbersome in specific patient groups, such as uncooperative children or bedridden individuals, due to the invasive nature of the corneal contact during measurements. Additionally, measurements can be influenced by the central cornea’s thickness [11,12,13,14].

The fluctuating nature of IOP, following a daily rhythm, suggests that measurements taken during normal activities or nocturnal periods could offer valuable insights for glaucoma management [15]. To monitor nychthemeral variations in IOP, patients are often hospitalized to facilitate measurements every 2–3 h over a full day. Despite the advent of innovative devices like implantable or portable tonometers [16,17], contact lens type [18,19], and self-operating rebound tonometers [20,21], there is an ongoing demand for tonometers that are simpler to use, more user-friendly, and suitable for everyday patient monitoring outside hospitals. The exploration of vibroacoustic properties as a means to ascertain pressure has been a focus of recent studies. Research has utilized less invasive acoustic wave vibrations to infer the biomechanical properties of the eye [22,23,24,25,26], finding correlations between IOP and parameters like corneal acoustic impedance [27], corneal wave speed [28,29], ocular resonance frequency [30,31], and vibration damping characteristics [32].

Our research team conducted a study utilizing vibroacoustic techniques to measure IOP [33]. The aim of that research was to identify vibroacoustic properties associated with changes in IOP and to propose a novel method for measuring IOP based on these properties. In experiments using ex vivo porcine eyeballs, it was discovered that both the resonance frequency and the change in the magnitude of the vibration response (CMVR) increased with rising IOP values, and a strong correlation between CMVR and IOP was established. However, this method, which involves fixing the eyeball in a jig for vibration application, presents challenges in adapting to in vivo IOP measurement techniques, necessitating further refinement.

In this paper, we conducted research with interest in the finding that the response that occurs when vibration is applied to the eye is related to changes in IOP. If vibration occurs while one object is attached to another object and measurement is performed, coupling between the two objects occurs and affects the measured vibration response. Here, we will look at contact stiffness and contact damping to indicate connectivity at the area where an object is in contact and find out how these values relate to actual IOP.

## 2. Materials and Methods

### 2.1. Specimen Preparation for Vibration Testing

In order to obtain fresh eyes with no changes in protein or tissue, we prepared six porcine eyes slaughtered on the same day at the Majang Livestock Market (Seoul, Republic of Korea). The prepared eyes were packed with ice packs in a Styrofoam box and transported to the laboratory without peribulbar fat removed to maintain humidity until the test began. Before starting the experiment in the laboratory, the remaining muscle and fat, except for the peribulbar tissue needed to fix the eye, were trimmed. To utilize the rebound tonometer, a thin layer of balanced salt solution (BSS) was applied to the eye to ensure continuous hydration. The methods performed in this study were conducted in compliance with relevant guidelines and regulations, and since the research used animal carcasses and did not involve humans or use living organisms, approval from the institutional animal care and use committee was not required.

### 2.2. Setting Up Vibration Experiment Equipment

**Figure 1:**
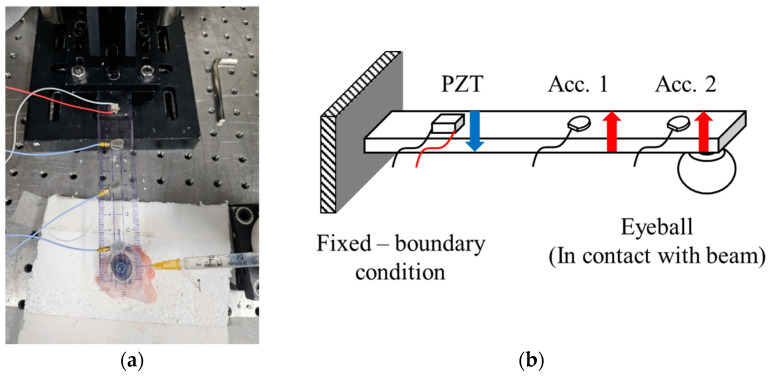
(**a**) Experimental setup to measure changes in vibration response according to pressure change; (**b**) schematic of vibration measuring equipment with fixed-free boundary condition.

In this study, to actually measure IOP, we adopted a method of placing a beam on the eyeball and then applying vibration. The contact stiffness that occurs at this time has a relatively low value compared to the beam or eye, so even if a difference occurs, it may be difficult to observe. Therefore, apart from the eyeball, which cannot arbitrarily adjust the stiffness, a plastic material with a low Young’s modulus was selected to lower the bending stiffness of the beam. Since the bending stiffness is also proportional to the cube of the thickness of the beam, a ruler was used to easily select the thinnest object possible. The method applied in this paper was designed to attach a plastic ruler to the pupil to aim for measurement in the frontal direction. Since a rebound tonometer is used to measure IOP, it is necessary to secure space in the pupil area. For this purpose, a small hole was drilled in the plastic ruler and the experimental settings were adjusted so that this part was located above the pupil, which can be seen in Figure 1. In order to apply vibration by changing the excitation force, a piezoelectric element actuator (PZT) was attached near the fixed end of the beam using epoxy resin. The PZT used was a PL055.3x (PICMA, Karsruhe, Germany), with an axial resonant frequency of >600 kHz and an operating voltage range of −20 to 100 V. To measure the vibration response and derive the transfer function, two accelerometers (Acc) were installed at different positions using mounting wax. The accelerometers were model 352A21/NC (PCB Piezotronics, Inc., Depew, NY, USA), featuring a sensitivity of 10.30 mV/g, a frequency range of 1.0 to 10,000 HZ, and a measurement range of ±500 g.

### 2.3. Experimental Method

To conduct experiments by varying the excitation force, the signal input to the PZT was set as follows. The magnitude of the voltage was set at two intensities, 0.4 V and 1 V, to obtain different excitation forces. A random signal capable of exciting a frequency band from 0 to 3600 Hz was applied for 30 s, which was designed to sufficiently converge when measuring the transfer function. Applied vibration is briefly described in Table 1. The transfer function of the acc to ACC was calculated using an ACC near the fixed end as a reference.

### 2.4. Transfer Function for Spectral Analysis of Linear Systems

In the field of vibration analysis, employing transfer functions for spectral analysis is very important. A transfer function serves as a mathematical representation of the relationship between an input signal (such as force or vibration response of transmission point) and an output signal (like acceleration or vibration response of observation point). This tool plays an important role in understanding how the system responds to external stimuli.

#### 2.4.1. Frequency Response Analysis

A transfer function describes how a system responds to inputs at various frequencies. These insights are critical to identifying properties such as natural frequencies, resonances, and damping characteristics.

#### 2.4.2. Input–Output Relationship

The transfer function details the transformation of an input signal as it traverses through the system. For instance, it can reveal the extent of amplification or attenuation at specific frequencies, providing a comprehensive understanding of the system’s behavior.

#### 2.4.3. Proportional Output in Linear System

In linear systems, the transfer function demonstrates that the magnitude of the output is directly proportional to the magnitude of the input. This linearity ensures that if the input signal is scaled up or down, the output will scale accordingly. This proportionality is vital in predicting system behavior under varying input conditions and is a cornerstone concept in linear vibration analysis.

In summary, the transfer function is an invaluable tool in spectral vibration analysis, offering a detailed perspective on the system’s frequency response and dynamic behavior in response to external inputs. In these instances, the equation of motion is simplified to that of the classical beam [34]

(1) $$D\frac{\partial^{4}w}{\partial x^{4}}+M\frac{\partial^{2}w}{\partial t^{2}}=0,$$

where *D* is bending stiffness per unit length (Nm^2^), w is beam deflection (m), *x* is coordinate of vibration measurement (m), *M* is mass inertia per unit length (kg/m), and *t* is time (s).

The harmonic solution of the beam deflection, the usual complex notation was used for displacement as

(2) wx,t=Rew^xeiwt,

where the circumflex (^) above w denotes a complex quantity. 

The satisfying beam function of the deflection is provided by

(3) $$\hat{w}\left(x\right)=A_{1}\sin\left(k_{b}x\right)+A_{2}\cos\left(k_{b}x\right)+A_{3}e^{k_{b}\left(x-L\right)}+A_{4}e^{-k_{b}x},$$

where $A_{n}\left(n=1,2,3,4\right)$ are the coefficients related to the boundary condition of the clamped-free beam, $k_{b}$ is the wavenumber related to the circular frequency, and *L* is the length of beam. The clamped-free beam has the following four boundary conditions:

(4) $$\hat{w}\left(0\right)=w_{0},\;\frac{\partial \hat{w}\left(0\right)}{\partial x}=0,\;\frac{\partial^{2}\hat{w}\left(0\right)}{\partial^{2}x}=0,\;\frac{\partial^{3}\hat{w}\left(0\right)}{\partial^{3}x}=0.$$

The following matrix system of equation is obtained from Equations (3) and (4) as

(5) $$\left[\begin{array}{cccc} 0 & 1 & e^{-k_{b}L} & 1\\ 1 & 0 & e^{-k_{b}L} & -1\\ -\sin k_{b}L & -\cos k_{b}L & 1 & e^{-k_{b}L}\\ -\cos k_{b}L & \sin k_{b}L & 1 & -e^{-k_{b}L} \end{array}\right]\left[\begin{array}{c} A_{1}\\ A_{2}\\ A_{3}\\ A_{4} \end{array}\right]=\left[\begin{array}{c} w_{0}\\ 0\\ 0\\ 0 \end{array}\right],$$

the equation is solved using symbolic calculations to obtain the transfer function of the beam and used in this study as

(6) $$\Lambda e^{i\Phi }=\frac{\hat{w}\left(x_{1}\right)}{\hat{w}\left(x_{2}\right)}=\frac{A_{1}\sin\left(k_{b}x_{1}\right)+A_{2}\cos\left(k_{b}x_{1}\right)+A_{3}e^{k_{b}\left(x_{1}-L\right)}+A_{4}e^{-k_{b}x_{1}}}{A_{1}\sin\left(k_{b}x_{2}\right)+A_{2}\cos\left(k_{b}x_{2}\right)+A_{3}e^{k_{b}\left(x_{2}-L\right)}+A_{4}e^{-k_{b}x_{2}}},$$

where Λ is the dimensionless amplitude and Φ is the phase of the transfer functions between the two different deflections at coordinates.

**Figure 2:**
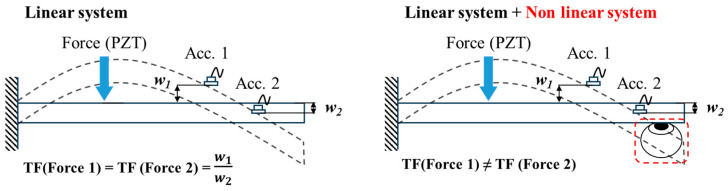
Schematic representation of transfer function analysis concept in both linear and non-linear systems with various inputs.

The characteristics of the transfer function presented above are typical for vibrations in a linear system. In this study, we aim to measure changes in IOP using the relationship between the experimental tendency of transfer function derived from a contact method with clamped-free boundary conditions. When linear system is actuated with different level of force, the transfer function response of the system remains the same. However, when a non-linear system, such as the eye, is introduced into a linear system, the overall system acquires nonlinear properties. Therefore, relationship between experimental result and linear system is analyzed to define the feasibility of estimating the IOP. Non-linear system exhibits diverse responses to variation in the applied force, which can elucidate the experimental tendency defining the relationship between IOP and transfer function response. Figure 2 provides a schematic representation that visually summarizes the discussed concepts and illustrates the system’s behavior in a straightforward manner

### 2.5. Methodology for Univariate Regression Analysis

#### 2.5.1. Overview of Univariate Regression Analysis

In our study, we conducted univariate regression analysis to model and analyze the relationship from experiment responses measured in the beam with non-linear system, such as porcine eyeball, under varying applied force. Using univariate regression analysis, we aim to predict IOP as a dependent variable and define independent variable from the experimental responses obtained from transfer function analysis. Univariate regression analysis is a simple yet powerful statistical method used to assess the impact of one variable’s changes on another. This approach is particularly valuable when aiming to understand how one variable influences another.

#### 2.5.2. Application of GLM for Univariate Analysis

GLM offers the capability to fit an appropriate regression model to the distribution of a specific variable. In the context of univariate analysis, it primarily focuses on modeling the changes in the dependent variable with respect to a single independent variable. By employing GLM, we were able to construct a regression model that suited the characteristics of our univariate data, accurately assessing the impact of the independent variable on the dependent variable.

This approach prioritizes understanding simple yet clear relationships without the complexity of considering multiple variables. Thus, univariate regression analysis proves to be an invaluable tool in exploring and predicting relationships between specific variables. In our study, we employed linear regression to model the relationship between a single independent variable and a dependent variable. The regression model is defined by the equation as.

(7) $$Y=\beta_{0}+\beta_{1}X+\varepsilon ,$$

where *Y* represents the dependent variable, *X* denotes the independent variable, $\beta_{0}$ is the intercept of the regression line, $\beta_{1}$ is the slope indicating the effect of *X* on *Y*, and ε is the error term, normally distributed with mean zero and variance $\sigma^{2}$. This equation provides a framework to analyze how changes in *X* influence the expected value of *Y*. In the context of GLMs with normal distribution, maximum likelihood estimation (MLS) aims to find parameter values that maximize the likelihood of observing the provided data. The likelihood function for the normal distribution is based on the probability density function of the normal distribution

(8) $$L\left(\beta_{0},\beta_{1},\sigma^{2}\right)=\prod_{i=1}^{n}\frac{1}{\sqrt{2\pi \sigma^{2}}}\exp\left(-\frac{{\left(Y-\left(\beta_{0}+\beta_{1}X\right)\right)}^{2}}{2\sigma^{2}}\right),$$

the estimation involves maximizing this function with respect to $\beta_{0},\beta_{1},$ and $\sigma^{2}$.

### 2.6. Application of Pearson Correlation in Univariate Analysis

#### 2.6.1. Overview of Pearson Correlation

In our research, to explore the feasibility of measuring the IOP, we conducted univariate analysis to derive the relationship of IOP and experiment frequency responses. Pearson correlation was employed to examine the linear relationship between two variables, predicted IOP and experiment frequency responses, in a univariate context. Pearson correlation is a widely used statistical method that quantifies the degree of linear correlation between two variables. It is particularly effective in identifying the strength and direction of a linear relationship, making it an essential tool in preliminary data analysis. 

#### 2.6.2. Pearson Correlation Coefficient

The Pearson correlation coefficient, denoted as “r”, ranges from −1 to +1. This coefficient measures the strength and direction of the linear relationship between variables. A coefficient close to +1 indicates a strong positive linear correlation, suggesting that as one variable increases, the other variable also increases. Conversely, a coefficient close to −1 indicates a strong negative linear correlation, where one variable increases as the other decreases. A coefficient around 0 signifies no linear correlation between the variables.

#### 2.6.3. Significance of Pearson Correlation

In our study, the Pearson correlation was pivotal in assessing the initial linear associations between variables. This analysis allowed us to identify potential relationships worthy of further investigation using more complex statistical methods. By quantifying the linear relationships between variables, Pearson correlation provided a foundational understanding that guided subsequent analyses and hypothesis formulation. In our analysis, we utilized Pearson correlation to assess the linear relationship between two variables. This coefficient is calculated using the formula as

(9) $$r=\frac{n\left(\sum ab\right)-\left(\sum a\right)\left(\sum b\right)}{\sqrt{\left[n\sum a^{2}-{\left(\sum a\right)}^{2}\right]\left[n\sum b^{2}-{\left(\sum b\right)}^{2}\right]}},$$

where *n* is the number of observations, and *a* and *b* are the observed values of the variables.

## 3. Results

**Figure 3:**
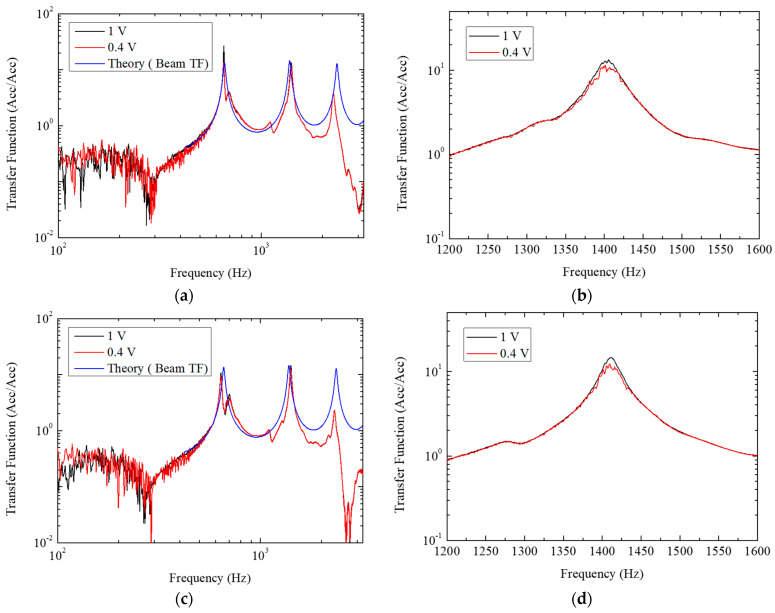
(**a**) The vibration responses of Eye 1 with frequency range from 100 to 3200 Hz; (**b**) close–up view of Eye 1′s vibration responses in the frequency range from 1200 Hz to 1600 Hz with a prominent peak observed around 1400 Hz; (**c**) the vibration responses of Eye 2 with frequency range 100 to 3200 Hz; (**d**) is the close–up view of Eye 2′s vibration responses in the frequency range of 1200 Hz to 1600 Hz.

As observed in Figure 3a,c, the experimental responses from various input voltages show similarities with the theoretical vibration mode response calculated from the classic beam. The profiles of the vibrational responses also exhibit remarkably similar shapes across the different porcine eyeballs. The PZT employed in the study was a lightweight and compact model designed for application on a plastic ruler, which accounts for the relatively higher target frequency range. The sub-700 Hz range did not generate sufficient excitation due to the physical characteristics of the system, resulting in responses that were predominantly noise-like. Beyond this range, the response resembled the typical shape of a transfer function. The similarity in vibrational response profiles, despite variations in eyes, suggests a low contact stiffness between the beam and the eyeball, indicating that the beam and the eyeball did not function as a single integrated system. This inference is further supported by the lack of shift in the peak frequency components, implying that the contact stiffness was significantly lower than the bending stiffness.

Further analysis using Figure 3b,d reveals that varying the excitation voltage from 1 V to 0.4 V does not significantly alter the frequency components, thereby rendering the differentiation of IOP based on this measure challenging. However, contact damping exhibited a meaningful difference, particularly in the magnitude at the peak. It is also noteworthy that the graph for the 1 V signal is considerably smoother compared to the 0.4 V signal, which appears jagged as if affected by noise. This nonlinear vibration phenomenon is attributable to the absence of rapid detachment and reattachment of contact.

**Figure 4:**
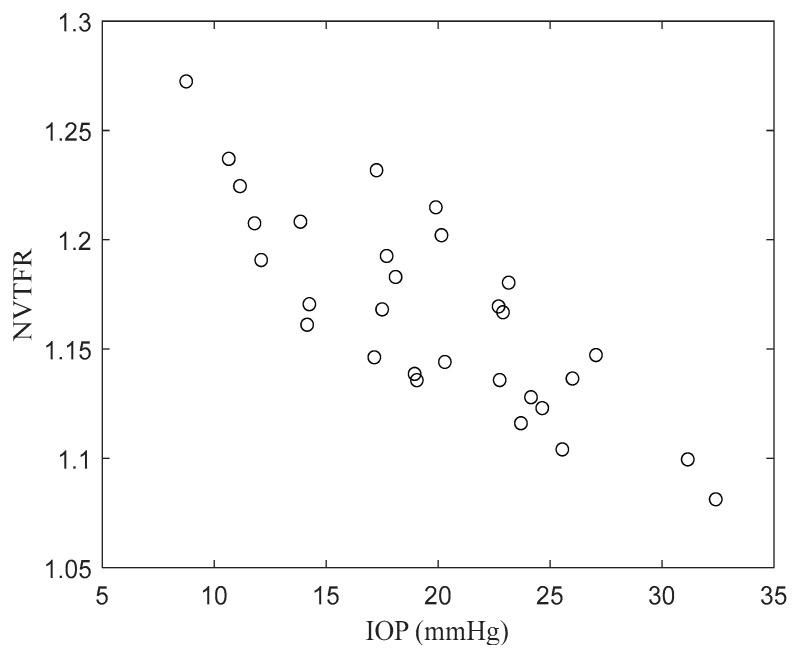
NVTFR in relation to the IOP.

The phenomenon of decreased peak frequency magnitude between 1200 Hz and 1600 Hz, when the excitation voltage increases from 0.4 V to 1 V, was observed across the entire eyeball. To quantify the ratio of this difference, a nonlinear vibration transfer function ratio (NVTFR) was calculated by dividing the peak magnitude at 1 V by that at 0.4 V.

(10) $$\xi =\frac{\Lambda \left(f_{1v}\right)}{\Lambda \left(f_{0.4v}\right)},$$

where ζ represents the NVTFR, $\Lambda \left(f_{1V}\right)$ is the transfer function magnitude of 1 V excitation responses at peak frequency between 1200 Hz and 1600 Hz. In Table 2, precise values can be found. Figure 4 displays the variation of the NVTFR with changes in the IOP for five different pressures across six individual eyeballs. The NVTFR values exhibit a tendency to decrease with increasing IOP.

Our regression analysis is indicative of employing a GLM to statistically interpret the relationship between IOP and NVTFR. Table 3 presents the results of the GLM regression analysis for NVTFR. The interpretation of these results reveals a highly significant correlation, as indicated by a *p*-value lower than 0.001, suggesting a close association between NVTFR and the measured outcomes.

Additionally, by utilizing the Pearson correlation coefficient, the correlation between NVTFR and the measured IOP was found to be −0.8111. This indicates a strong negative correlation between the two variables.

This section may be divided by subheadings. It should provide a concise and precise description of the experimental results, their interpretation, and the experimental conclusions that can be drawn.

## 4. Discussion

Random vibrations induced by PZT excite the beam across the entire frequency band. This excitation, spanning the whole frequency spectrum, influences the frequency responses, contingent upon factors such as the object’s shape, material, added mass, and boundary conditions. From the beam’s perspective, while its shape, material, and added mass remain constant, the only variable is the boundary condition at the free end interfacing with the porcine eye. The contact with the eyeball at this free end, excited by the beam’s vibrations, results in altered frequency responses due to the coupling effect between the two entities. By analyzing the frequency response ratio measured in the beam with varying applied force, the dynamic characteristics of porcine eyeball are defined based on experimental tendencies. Consequently, one can discern dynamic characteristics such as stiffness and damping.

A change in stiffness signifies a shift in the frequency range where the signal’s primary energy resides. Typically, an increase in a structure’s stiffness elevates its system’s frequency components, whereas a decrease in stiffness leads to a corresponding reduction in these components. In standard spectrum analysis, frequencies significantly higher relative to surrounding components are identified as natural frequencies. However, in this study, which employs the transfer function between ACC signals, these peaks are not natural frequencies but merely peak frequencies. The values represented by these frequency components lack inherent physical meaning.

When variations arise from damping, a distinct shift is noticeable in the magnitude at the defined peak frequency. A significant reduction in magnitude, coupled with a peak that diminishes gradually and extensively rather than abruptly, denotes substantial damping. Conversely, minimal damping exhibits an opposite trend. Similar to stiffness, the damping values derived from the transfer function in this research lack direct physical interpretation. Nevertheless, they offer insights into the damping tendencies.

The contact stiffness between the beam and the eyeball, when vibrated in contact, is sufficiently large; the resulting response will be identical to that of the beam and eyeball vibrating as a single entity. However, the method devised in this study involves the beam being in barely contact with the eyeball, implying that during actual vibration, the two bodies will repeatedly attach and detach. The vibration patterns when they are separated and attached differ, and this movement, acting as an excitation, continually occurs, ultimately inducing a coupling effect and leading to nonlinear vibrations. Additionally, the eyeball, being a viscoelastic material whose characteristics vary with IOP, further intensifies this nonlinearity. Changes in the magnitude of the induced vibrations will alter the coupling effect at the contact point between the beam and the eyeball.

**Figure 5:**
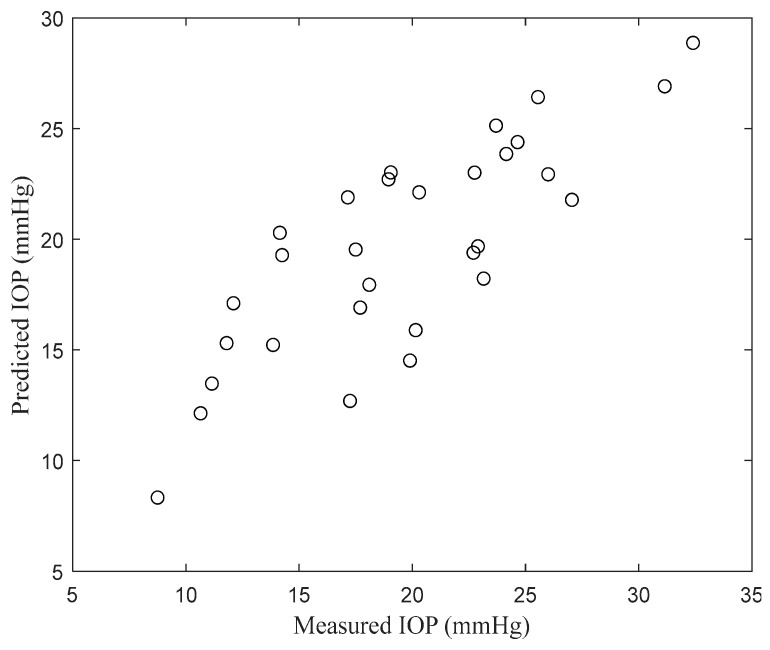
The measured IOP against the predicted IOP by GLM.

To predict IOP, a formula enabling the substitution of NVTFR can be derived by applying the results from Table 3 to Equation (7).

(11) $$IOP_{P}=145.0734+\left(-107.4694\times \zeta \right),$$

where $IOP_{P}$ is predicted IOP. The results of applying NVTFR to the derived GLM model and its prediction compared with the measured IOP are illustrated in Figure 5. This figure displays the outcomes, showcasing the relationship between the predicted values using the GLM model and the actual IOP measurements.

Our study demonstrates a significant breakthrough in measuring IOP by employing a beam-shaped experimental device with applied vibration. The use of nonlinear vibration, coupled with transfer function analysis, not only sets our approach apart from conventional methods but also opens up new avenues for non-invasive IOP measurement. This method could potentially revolutionize the standard practices in ophthalmology by offering a safer and more comfortable alternative for patients.

In our study, the NVTFR emerged as a crucial indicator in relation to IOP. NVTFR values exhibit a tendency to decrease with increasing IOP across different pressure conditions. This trend suggests a close association between NVTFR and IOP.

To statistically interpret the relationship between IOP and NVTFR, GLM has been used. According to the GLM regression analysis results presented, NVTFR shows a highly significant correlation with the measured outcomes, evidenced by a *p*-value lower than 0.001.

**Figure A1:**
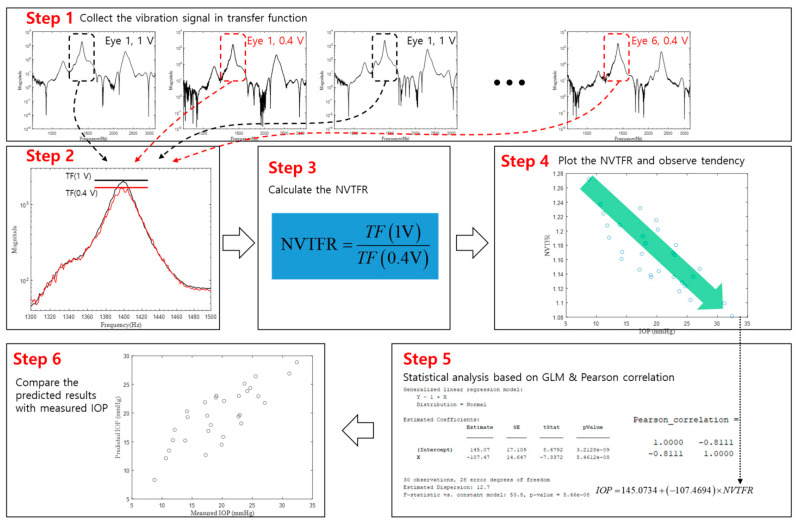
Visual representation of research methods and procedures.

Furthermore, the analysis utilizing the Pearson correlation coefficient also revealed a strong negative correlation between NVTFR and the measured IOP, with a coefficient of −0.8111. This indicates a strong inverse relationship between the two variables. Such findings suggest that NVTFR could serve as an important indicator in IOP measurements, providing a significant benchmark for future research and applications. See Appendix A, Figure A1 for the whole flow of this study.

The collaboration between mechanical engineering and ophthalmology in this study exemplifies the benefits of interdisciplinary research. By applying mechanical principles to a medical problem, we have successfully demonstrated how technological innovation can lead to significant improvements in medical diagnostics. This integration paves the way for future collaborative efforts that can lead to further advancements in medical technology.

The non-invasive nature of our methodology is its most notable strength, making it an appealing alternative to more invasive traditional IOP measurement techniques. However, the limitations of our study, such as the lack of human eye trials and a limited sample size, highlight areas for improvement. While our findings are promising, they must be interpreted with an understanding of these constraints. The limited sample size, in particular, raises questions about the generalizability of our results, and future studies with a larger and more diverse sample are essential to validate our findings.

Although the direct application of our findings in a clinical setting may not be immediately feasible, our study has important implications for the future of patient care in ophthalmology. The prospect of a non-invasive, patient-friendly IOP measurement technique is particularly exciting. It holds the potential to improve patient compliance and comfort, especially among populations that are more sensitive to invasive procedures, such as children or those with pre-existing eye conditions.

Looking ahead, our research aims to extend the application of this technique to human eyes. Conducting trials on human subjects will be a critical step in moving from theoretical validation to practical application. The ultimate goal of developing a method for measuring IOP through the eyelid, without direct contact with the eye, could redefine the standards of eye care. This would not only enhance the patient experience but could also lead to more frequent and accurate IOP measurements, thereby improving the management of conditions like glaucoma.

Our study lays the foundation for a new paradigm in IOP measurement. The journey from conceptualization to implementation will require further research, innovation, and interdisciplinary collaboration. However, the potential impact on patient care and ophthalmological practice makes this a worthy endeavor, marking a significant step forward in the quest for better eye care solutions.

## Figures and Tables

**Table 1:** Specification of vibration.

Input Voltage (V)		Signal Type	Frequency Range (Hz)	Averaging Time (s)
0.4	1	Random	0~3200	30

**Table 2:** Measured IOP values by individual eyes.

Experimental Case	IOP (mmHg)			Vibration Experiment Outcomes		
	Initial	Final	Average	$\Lambda \left(f_{1V}\right)$	$\Lambda \left(f_{0.4V}\right)$	NVTFR
Eye 1	17.4	17.1	17.25	2025.982	1644.741	1.2318
	25.1	22.3	23.7	2024.131	1813.608	1.1161
	20.1	18.0	19.05	2113.224	1860.628	1.1358
	26.7	22.6	24.65	2052.942	1828.111	1.1230
	14.3	13.4	13.85	2047.114	1674.261	1.2083
Eye 2	20.2	19.6	19.9	2461.591	2026.280	1.2148
	9.6	7.9	8.75	2450.163	1925.601	1.2724
	12.2	10.1	11.15	2494.068	2036.790	1.2245
	24.2	21.3	22.75	2464.795	2170.038	1.1358
	21.3	19.0	20.15	5461.470	2047.716	1.2021
Eye 3	32.3	30.0	31.15	2174.268	1977.458	1.0995
	34.0	30.8	32.40	2231.677	2063.924	1.0813
	27.8	24.2	26.00	2291.617	2016.355	1.1365
	24.2	21.6	22.90	2258.818	1935.864	1.1668
	21.6	13.8	17.70	2324.023	1948.758	1.1926
Eye 4	29.0	25.1	27.05	2166.710	1888.592	1.1473
	25.1	20.3	22.70	2159.476	1846.510	1.1695
	20.3	15.9	18.10	2167.298	1832.082	1.1830
	20.9	17.0	18.95	2118.316	1860.406	1.1386
	22.3	18.3	20.30	2095.362	1831.448	1.1441
Eye 5	26.1	20.2	23.15	2263.755	1917.842	1.1804
	25.2	23.1	24.15	2281.210	2022.404	1.1280
	15.1	13.2	14.15	2213.719	1906.526	1.1611
	17.2	17.1	17.15	2163.504	1887.492	1.1462
	13.1	11.1	12.10	2162.250	1815.901	1.1907
Eye 6	32.2	18.9	25.55	2155.994	1952.718	1.1041
	18.9	16.1	17.50	2115.020	1810.555	1.1682
	16.1	12.4	14.25	2190.454	1871.339	1.1705
	12.4	11.2	11.80	2172.288	1798.966	1.2075
	11.2	10.1	10.65	2137.333	1727.825	1.2370

IOP = intraocular pressure; $\Lambda \left(f_{nV}\right)\left(n=1,0.4\right)$ = Peak magnitude frequency range between 1200 Hz and 1600 Hz; NVTFR = Nonlinear Vibration Transfer Function Ratio.

**Table 3:** Estimated results and regression coefficients regarding NVTFR in GLM.

Fixed Effects	Estimate	SE	*p*-Values
Intercept	145.0734	17.109	<0.001
NVTFR	−107.4694	14.647	<0.001

GLM = Generalized Linear Model; NVTFR = Nonlinear Vibration Transfer Function Ratio.

## Data Availability

Data are available upon reasonable request.
